# Pinoresinol stimulates keratinocyte proliferation and downregulates TNF‐α secretion in peripheral blood mononuclear cells: An experimental in vitro study

**DOI:** 10.1002/hsr2.998

**Published:** 2022-12-16

**Authors:** Elias Haapakorva, Hannu Raunio, Atte von Wright, Ilkka Harvima

**Affiliations:** ^1^ Kasve Ltd Kuopio Finland; ^2^ School of Pharmacy University of Eastern Finland Kuopio Finland; ^3^ Institute of Public Health and Clinical Nutrition University of Eastern Finland Kuopio Finland; ^4^ Department of Dermatology University of Eastern Finland and Kuopio University Hospital Kuopio Finland

**Keywords:** cytokines, inflammation, lignans, plant resins, wound healing

## Abstract

**Background and Aims:**

Natural coniferous resins are used in traditional medicine for the treatment of skin wounds. Coniferous wood resins (“callus” resin) are a mixture of abietic (resin) acids, lignans such as pinoresinol, and *p*‐coumaric acid. The wound‐healing properties of resins are thought to be related to their antimicrobial properties, but also to their effects on cell proliferation and inflammation. The purpose of this study was to identify and investigate the effects of novel aqueous dispersions of resin and its main components in the proliferation of human primary keratinocytes in vitro and in the expression of proinflammatory cytokines in human peripheral blood mononuclear cells.

**Methods:**

The proliferation studies were performed under low and high calcium conditions with or without added growth stimulators at the time points of 2 and 6 days using AlamarBlue Cell Viability Reagent. The cytokine release assay was carried out by incubating the cells with the test articles for 18 h, after which the levels of tumor necrosis factor‐α (TNF‐α), interleukin‐1β (IL‐1β), IL‐6, and IL‐8 were measured in the supernatant by enzyme‐linked immunosorbent assay.

**Results:**

Resin and the purified lignan PINO, but not *p*‐coumaric acid or abietic acid (industrial tall oil rosin), enhanced the proliferation of human keratinocytes in vitro and inhibited the expression of TNF‐α, and to a lesser extent the expression of IL‐1β in peripheral blood mononuclear cells.

**Conclusions:**

In this study, novel aqueous dispersions of spruce resin were used to investigate the effects of main resin components on keratinocyte proliferation and on the expression of key proinflammatory cytokines known to be associated with chronic wounds. The observations suggest that lignans, such as PINO, but not resin acids, are the components of resins that mediate the proliferative and TNF‐α‐suppressing effects. Lignans including PINO were identified as novel potential compounds in the treatment of chronic skin ulcers.

## 1 INTRODUCTION

Natural coniferous “callus” resins collected from injured Norway spruce trees (*Picea abies* (Linnaeus, 1753; Karsten, 1881) have been used in traditional medicine, particularly in Northern Europe, as a self‐remedy in various skin diseases, particularly skin wounds. Wood resins are complex mixtures of several compounds. Of wood resins, the wood oleoresins and rosins extracted industrially from coniferous tall oil are composed exclusively of terpenoid resin acids, mainly abietic acid. The “mature” wood resins from injured trunks of coniferous trees (“callus resin”) contain, in addition to resin acids, lignans and *p*‐coumaric acids (PCAs),^1^, ^2^ which are secreted slowly from wood cells in the tree bark and mixed with the oleoresin.

Formulated as salves or ointments, wood resins and tall oil rosin are antimicrobial against a wide range of bacteria, fungi, and yeasts.^2^, ^3^, ^4^ In human clinical trials, salves prepared from wood “callus” resins also improve the healing of skin ulcers,^5^, ^6^, ^7^ indicating that their ingredients enhance skin renewal, possibly by an unspecific mechanism involving the expression of cytokines and growth factors in skin tissues.

In wood resins, lignans and coumaric acid are water‐soluble, whereas the terpenoid rosins are, in general, poorly soluble in water,^8^ making their investigations difficult in in vitro tests. Novel methodologies allow, however, the preparation of stable aqueous dispersions or emulsions of natural wood rosins. These aqueous solutions contain, in addition to lignans and coumaric acid, small amounts of water‐soluble oxidized resin acids.^2^ These aqueous dispersions allow reliable in vitro laboratory testing not amenable to salves or ointments. In earlier studies, the novel water dispersions of natural wood resins were shown to be as antimicrobial as salves or ointments, suggesting that the resin acids in both wood resins and tall oil rosins are the components responsible for the antiseptic property of the resins in general.^2^

Pinoresinol (PINO), among other lignans, belongs to phytoestrogens that occur naturally in plants and are structurally similar to mammalian estrogens. Although scientific interest in lignans has increased significantly in recent years, very little is known about their possible pharmacological properties in humans. However, it has recently been reported that PINO promotes keratinocyte re‐epithelialization^1^ and osteoblast proliferation^9^ in an in vitro wound healing assay.

The purpose of this study was to identify the resin substances that might play a role in wound healing. In this investigation, we evaluated how the aqueous dispersions of coniferous wood resins (RE) of natural “callus” origin and industrial rosin (RO) originating from tall oil influence the proliferation of skin keratinocytes in vitro and whether they influence the expression of cytokines in human blood cells. The test articles were water dispersions of RE and RO, with rapeseed oil (RY) as a cosolvent. In addition, the tests were performed with water solutions of purified PINO, secoisolariciresinol (SECO), and nortrachelogenin (NTG). PINO and SECO are among the most abundant lignans in RE, whereas NTG is a common lignan found in knots (branch bases), but to a lesser extent in resins of *P. abies*.^10^ The test articles also included purified PCA, an abundant component in RE, but absent in RO.

## 2 MATERIALS AND METHODS

### 2.1 Test articles: Aqueous dispersions of rosins, lignans, and PCA

RE is an aqueous dispersion prepared from “callus” resin. Shortly, callus resin collected from trunks of Norway spruce (*P. abies*) was dissolved in RY, glycerol (GLY), or 1,3‐propanediol (DI) and transferred further to saline to reach a stable oil‐in‐water emulsion.^2^ RO was prepared similarly by using the tall oil rosin instead of the “callus” resin. These dispersions were used at various dilutions in the experiments. Identical solvent controls, prepared without RE or RO, were used as controls.

The composition of RE and RO dispersions was analyzed using short‐column gas chromatography‐flame ionization detection and gas chromatography/mass spectrometry (GC‐MS). Component identification was based on retention times and mass spectra (GC‐MS) and the quantification was made on short‐column GC with betulinol as standard as previously described.^2^

The RE dispersion was a mixture of oxidized resin acids, lignans, and PCA. One main component of lignans was PINO, comprising approximately 42 of all lignans present in RE. The RO dispersion was composed of oxidized resin acids only, and lignans, PCA, and the main resin acids were absent. In the experiments, RO was further diluted to correspond to the oxidized resin acids content of RE.

The aqueous solution of purified PCA (Sigma‐Aldrich; catalog number 501‐98‐4) was prepared by dissolving the compounds in saline and further diluted to RY at the same concentrations as in the RE solution.

PINO, SECO, and NTG were obtained as 100× dimethyl sulfoxide stock solutions (Oy Separation Research Ab). PINO and SECO were diluted further to RY to reach a final concentration of 90 ppm (PINO) and 24 ppm (SECO) to match the concentration measured in the RE solution. NTG, which is abundant in branch knots but is sparse in callus resin, was diluted to a final concentration of 50 ppm to match the range of PINO and SECO. The test articles used in this study are listed in Table 1.

**Table 1:** List of test articles and the dilutions used

Test article	RE^a^	RO^a^	PCA^b^	PINO^b^	SECO^b^	NTG^c^
PCA	247 ppm	‐	250 ppm	‐	‐	‐
Oxidized resin acids	150 ppm	145 ppm	‐	‐	‐	‐
Lignans (all)	210 ppm	‐	‐	‐	‐	‐
>PINO	88 ppm	‐	‐	90 ppm	‐	‐
>SECO	24 ppm	‐	‐	‐	24 ppm	‐
NTG	‐	‐	‐	‐	‐	50 ppm

Abbreviations: GC‐MS, gas chromatography/mass spectrometry; NTG, nortrachelogenin were prepared the same way as RE; PCA, *p*‐coumaric acid; PINO, pinoresinol; RE, dissolved callus resin dispersed in rapeseed oil and saline 1:1; RO, rosin; SECO, secoisolariciresinol.

^a^ Quantified using GC/MS.

^b^ Diluted to correspond to the measured quantities in RE.

^c^ Diluted to match the range of SECO and PINO.

### 2.2 Cell viability assays

Cell viability assays were conducted to assess the effect of test articles in the vehicles at two different concentrations, 10 and 1. Primary normal human epidermal keratinocytes, adult (HEKa) (Thermo Fisher; catalog number C0055C) were seeded using supplemented medium 154 (Life Technologies; catalog number M154500) at 2 × 10^5^ cells/well and allowed to settle for 1 h. Cells were then treated with the test articles/vehicles, negative controls (medium and 10 phosphate‐buffered saline [PBS]). At 20 h posttreatment, AlamarBlue Cell Viability Reagent (Life Technologies; catalog number DAL1025) was added to each well, and 4 h later, the cell viability was assayed from triplicate wells by reading fluorescence intensity of AlamarBlue reagent on a fluorescence plate reader according to the manufacturer's instructions.

The test articles and controls were prepared as follows: dilutions of 1:10 (10) or 1:100 (1) of the test articles were made directly in the well by adding either 20 or 2 µl of the test articles to an appropriate amount of medium to reach the final volume of 200 µl in the well. The 1:10 and 1:100 dilutions of vehicles and saline controls were prepared in a similar fashion.

### 2.3 Keratinocyte proliferation assays

Human keratinocytes were isolated from the foreskins of one healthy infant donor undergoing circumcision.^11^ Keratinocytes were cultured with a standard method using a medium containing Keratinocyte‐SFM™ (KSFM) (Life Technologies), epidermal growth factor (EGF) (5 ng/ml), bovine pituitary extract (BPE) (50 μg/ml), penicillin (100 U/ml), and streptomycin (100 μg/ml). The cells were seeded into flasks at a density of about 10,000–20,000 cells/cm^2^. The flasks were incubated in 5% CO_2_ at 37°C. The medium was changed every 2–3 days. After the primary culture, cells were passaged every 3–6 days and used between passages 3 and 8.

One day before the experiment, the cells were plated in wells of 96‐well plates at 20,000 cells/well in a volume of 200 μl of medium and incubated overnight at 5% CO_2_ at 37°C. After incubation, the cell culture medium was replaced with 200 μl/well of complete or basal (without EGF and BPE) KSFM.

A parallel experiment under high calcium conditions was performed by using Dulbecco's modified Eagle's medium (DMEM) (Life Technologies), with or without added serum (10 fetal calf serum (FCS); Biological Industries), supplemented with 100 U/ml penicillin and 100 μg/ml streptomycin. High‐calcium conditions induce differentiation and epithelium development of monolayer keratinocytes, and DMEM is a well‐characterized medium for culturing and preparing keratinocyte epithelium.^12^

In KSFM, EGF and BPE act as growth stimulators, and in DMEM, FCS stimulates cell growth. Without these growth stimulators, the activation of slow‐growing cells may be better seen, whereas the inhibition of well‐growing cells may be observed in conditions with these additives.

The cells were treated with the test compounds RE, RO, PINO, or PCA and the cell proliferation was measured at the time points of 2 and 6 days using AlamarBlue Cell Viability Reagent (Invitrogen) according to the manufacturer's instructions. The experiments were performed using five parallel wells.

### 2.4 Cytokine release assay

Human whole blood was obtained from two healthy human subjects (males aged 45 years and females aged 42 years) using heparin‐coated vacutainers. Peripheral blood mononuclear cells (PBMCs) were immediately isolated from whole blood using a standard density separation protocol (Histopaque 1077; Sigma). Cell viability was assessed by Trypan Blue exclusion and was found to be >99 before transfer to plates. PBMCs were seeded in wells of a 12‐well cell culture plate at a density of 500,000 cells/ml in RPMI‐1640 medium supplemented with 10 FCS. Cells were allowed to equilibrate in a humidified cell culture (37°C, 5% CO_2_) incubator for 30 min before the addition of the test articles.

The test articles were added to the wells at the final concentration of 4 or 1. The vehicle (RY) was used to equalize the volume of test article/vehicle added to each well. Beclomethasone, a well‐characterized glucocorticoid, was used as a reference compound. Glucocorticoids are corticosteroids that upregulate the expression of anti‐inflammatory proteins in the nucleus and repress the expression of proinflammatory proteins.^13^ Beclomethasone was prepared as a concentrated stock solution and was diluted in RPMI medium to a final concentration of 10 μM. The test articles were: Resolain™ (Repolar Pharmaceuticals Ltd), a commercially available scalp tonic containing resin acids, RE, RO, PINO, and PCA. Resolain™ contains resins at approximately 500‐fold concentration compared to RY‐based test solutions.

Unstimulated and stimulated control wells were treated identically, resulting in a final concentration of four vehicles across the plates.

PBMCs were preincubated with the test articles for 60 min before stimulation with lipopolysaccharide (LPS) (*Escherichia coli* O55:B55; Sigma). LPS solution was prepared in RPMI cell culture medium and added to appropriate wells to achieve a final concentration of 100 ng/ml. Unstimulated control wells received an identical volume of RPMI medium at this point. The plates were centrifuged (300*g* for 3 min) to pellet the cells 18 h after the addition of LPS, and the supernatant was collected for cytokine analysis. The following cytokines were measured in the supernatant by enzyme‐linked immunosorbent assay: tumor necrosis factor‐α (TNF‐α), interleukin‐1β (IL‐1β), interleukin‐6 (IL‐6), and interleukin‐8 (IL‐8).

### 2.5 Group definition

The cell viability assays with keratinocytes were performed with 1 and 10 RE, RY, and PBS. The initial keratinocyte proliferation assays included the lignans PINO, SECO, and NTG, in addition to the RE, RO, and PCA at concentrations of 0, 5, 1, 2, and 4. These experiments were performed in high‐calcium conditions (DMEM) with added growth stimulators (FCS) only to screen the test articles for further experiments. In the following keratinocyte proliferation assays, the test articles were tested in high and low calcium conditions, with or without added growth stimulators, for 2 or 6 days. The concentrations used were the same as in the initial screening. The cytokine release assays were performed using the same set of test articles as keratinocyte proliferation assays, supplemented with commercially available Resolain™ scalp tonic, which was used as a control solution for RE. The different test groups and testing conditions are summarized in Table 2.

**Table 2:** Group definitions and concentrations (%) used

Assay	RY	RE	RO	PCA	PINO	SECO	NTG	Resolain
Keratinocyte viability assay	1, 10	1, 10	‐	‐	‐	‐	‐	‐
Proliferation assay, high Ca, without growth stimulators	‐	0.5–4	0.5–4	0.5–4	0.5–4	0.5–4	0.5–4	‐
Proliferation assay, high Ca, with growth stimulators	‐	0.5–4	0.5–4	0.5–4	0.5–4	0.5–4	0.5–4	‐
Proliferation assay, low Ca, without growth stimulators	‐	0.5–4	0.5–4	0.5–4	0.5–4	0.5–4	0.5–4	‐
Proliferation assay, low Ca, with growth stimulators	‐	0.5–4	0.5–4	0.5–4	0.5–4	0.5–4	0.5–4	‐
Cytokine release assay in PBMCs	‐	1, 4	1, 4	1, 4	1, 4	‐	‐	1, 4

Abbreviations: NTG, nortrachelogenin were prepared the same way as RE; PBMC, peripheral blood mononuclear cell; PCA, *p*‐coumaric acid; PINO, pinoresinol; RE, dissolved callus resin dispersed in rapeseed oil and saline; RO, rosin; RY, rapeseed oil dispersed in saline; SECO, secoisolariciresinol.

### 2.6 Statistical analyses

In tests on cell viability and in proliferation assays, mean (±SD) values of replicates from parallel wells were calculated and used for statistical analysis. Three biological replicates were used in the cell viability assay and five biological replicates were used in proliferation assays. One‐way analysis of variance (ANOVA) with multiple comparisons (Dunnett's test) was used to compare the vehicle control (RY) and the test article dilution groups, individually. When applicable, paired *t*‐test was performed to compare the effect of the test article on the corresponding vehicle group.

For the cytokine release experiments, the effect of test articles on cytokine release is expressed as a percentage of the vehicle control (RY). Data are expressed as the mean ± SD for three replicate wells and the statistical analysis between data sets was carried out by one‐way ANOVA followed by Dunnett's posttest.

Statistical analyses were carried out using GraphPad Prism software, version 9.3.1 (GraphPad Software Inc.). *p* Values of ≤0.05, ≤0.01, and ≤0.001 were considered statistically significant.

## 3 RESULTS

### 3.1 Keratinocyte viability in 24‐h tests

**Figure 1:**
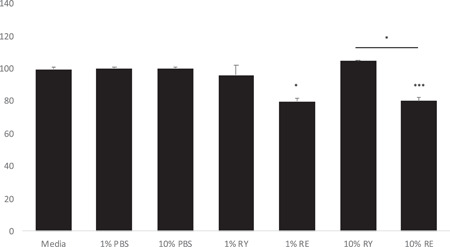
Effect of test article and cosolvents on cell viability in keratinocytes in 24‐h incubation in concentrations 1 or 10. Values represent means of three replicates ± SD. Statistical analysis between data sets was carried out by one‐way ANOVA followed by Dunnett's posttest. *p < 0.05 and ***p < 0.001 compared to media‐only or to corresponding vehicle control. ANOVA, analysis of variance; PBS, phosphate‐buffered saline; RE, dissolved callus resin dispersed in rapeseed oil and saline; RY, rapeseed oil dispersed in saline.

At a 10% concentration, RE significantly decreased keratinocyte viability at 24 h, whereas the control dispersions without RE, but with RY, did not have significant effects. Even at 1 concentration RE significantly decreased the cell viability compared to the media‐only control (Figure 1).

### 3.2 Keratinocyte proliferation in 2/6 days

**Figure 2:**
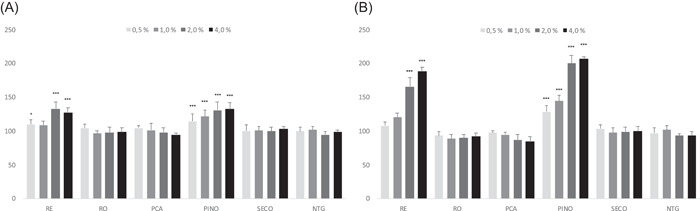
(A and B) Representative results of keratinocyte proliferation assays. The test articles were incubated with keratinocytes in high calcium conditions (DMEM) with added growth stimulators (FCS) for 2 (A) or 6 (B) days and the cell proliferation was measured using AlamarBlue assay. Data are expressed as percentages of the vehicle control group (RY). RE and PINO had a significant, dose‐dependent effect on keratinocyte proliferation, whereas other lignans did not. Values represent means of five replicates ± SD. Statistical analysis between data sets was carried out by one‐way ANOVA followed by Dunnett's posttest. ***p < 0.001 and *p < 0.05 denotes significant increase compared to the control. ANOVA, analysis of variance; DMEM, Dulbecco's modified Eagle's medium; FCS, fetal calf serum; RE, coniferous wood resins; RY, rapeseed oil; PINO, pinoresinol.

In proliferation experiments with high calcium conditions (DMEM), RE and PINO consistently and concentration‐dependently increased keratinocyte proliferation at both 2‐ and 6‐day time points, which may indicate that PINO is the main component in the resins modulating keratinocyte proliferation (Figure 2A,B). The same pattern was also observed in other tested conditions, which included both high and low calcium medium together with or without added growth stimulators, at the time points of 2 and 6 days. SECO, and to a lesser extent NTG, increased keratinocyte proliferation only in low calcium conditions at a 6‐day time point, but not in other tested conditions or time points. As expected, no consistent increase or decrease in proliferation was observed with RO or PCA (Supporting Information: Table 3).

### 3.3 Cytokine release from PBMCs

**Figure 3:**
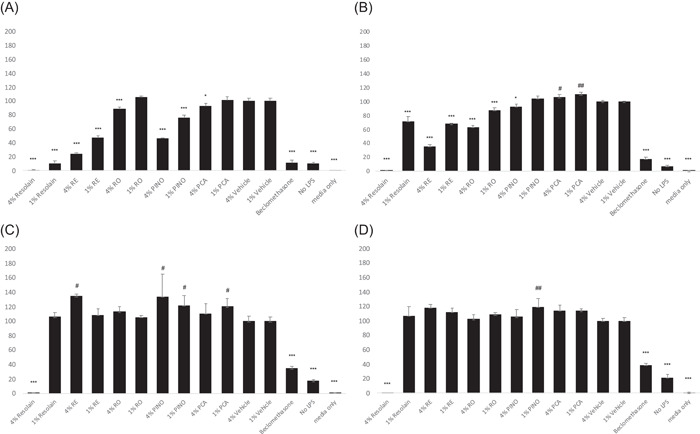
(A–D) Effect of the test articles on TNF‐α (A), IL‐1β (B), IL‐6 (C), and IL‐8 (D) release from human PBMCs from a 45‐year‐old male donor stimulated with LPS (100 ng/ml). Cytokine levels were measured at 18 h after the LPS induction. Data are expressed as the percentage of the vehicle control groups (cells receiving vehicle and LPS) and shown as mean ± SD from triplicate wells. The “no‐LPS” wells represent the basal release of cytokine from unstimulated cells. Statistical analysis between data sets was carried out by one‐way ANOVA followed by Dunnett's posttest. ***p < 0.001 and * *p* < 0.05 denotes significant cytokine release reduction compared to control. ^##^ *p* < 0.01 and ^#^ *p* < 0.05 denotes a significant elevation of cytokine release compared to the control. ANOVA, analysis of variance; TNF‐α, tumor necrosis factor‐α; IL‐1β, interleukin‐1β; LPS, lipopolysaccharide; PBMC, peripheral blood mononuclear cell.

The effects were similar with both donors. The results of the male donor are given here in detail as a representative example (Figure 3A,D), while those of the female donor are included in the Supporting Information: Figure 4A–D.

i. *Effect of the test articles on the release of TNF‐α from PBMCs*:
  Resolain™, RE, RO, PINO, and PCA induced a concentration‐dependent inhibition of TNF‐α release. RE, RO, PINO, and, to a lesser extent, PCA inhibited TNF‐α release at a concentration of 4. At the lower concentration of 1, only RE and PINO had a significant inhibitory effect. As expected, beclomethasone markedly inhibited TNF‐α release. Levels of TNF‐α in wells with medium only were essentially zero (Figure 3A).
ii. *Effect of the test articles on the release of IL‐1β from PBMCs*:
  Resolain™, RE, RO, and PINO induced a concentration‐dependent inhibition of IL‐1β release. RE, RO, and, to a lesser extent, PINO, inhibited IL‐1β release at the concentration of 4, whereas PCA did not. At the concentration of 1, only RE and RO inhibited IL‐1β release. As expected, beclomethasone markedly inhibited IL‐1β release and levels of IL‐1β in wells with medium only were essentially zero (Figure 3B).
iii. *Effect of the test articles on the release of IL‐6 from PBMCs*:
  RE and PINO appeared to enhance IL‐6 release at a concentration of 4. PINO also increased the release of IL‐6 at a concentration of 1. Only Resolain at a concentration of 4 inhibited IL‐6 release. Beclomethasone markedly inhibited IL‐6 release, and levels of IL‐6 in wells with medium only were essentially zero (Figure 3C).
iv. *Effect of the test articles on the release of IL‐8 from PBMCs*.

Resolain™ induced an inhibition of IL‐8 release at the concentration of 4. PINO induced a slight increase in the release of IL‐8 at the concentration of 1. Beclomethasone also inhibited the release of IL‐8 from stimulated PBMCs, and levels of IL‐8 in wells with medium only were essentially zero (Figure 3D).

## 4 DISCUSSION

### 4.1 Viability of keratinocytes within the first 24 h

The aim of the initial keratinocyte viability studies was to find out the possible effects of the different test articles on cell growth. The tests were performed with commercially available HEKa cells as they are well characterized and known to grow reliably. Of the present test articles, GLY and DI appeared to influence the keratinocyte viability whereas RY was tolerated well, and thus, RY was qualified as a cosolvent for the following experiments. The aqueous 10 and 1 RE dispersions with RY as the cosolvent decreased HEKa keratinocyte viability within 24 h, but only by 20 at both 1 and 10 RE concentrations. The measured decrease in cell number was not significant at 1 concentration compared with the vehicle.

Even though the slight decrease in cell viability is a confounder at the initial stages of keratinocyte cultures, the increase and preservation of high keratinocyte growth over 2–6 days indicate that the test articles enhance keratinocyte proliferation and that this enhancement overcomes the possible toxic effects on the cells at early stages of exposure. In addition, a general delay may occur before the positive influences on cell growth are observable after exposure to the test articles.

### 4.2 Keratinocyte proliferation in 2–6 days

The aqueous solutions with purified PINO and the RE dispersion containing PINO as the main lignan component, in addition to oxidized resin acids and PCA, enhanced keratinocyte proliferation in a dose‐dependent manner in the 2/6‐day experiments. This phenomenon was observed in all conditions tested, but it was not seen with the RO emulsion containing oxidized resin acids only, suggesting that the lignans are the likely ingredients enhancing keratinocyte proliferation (see Supporting Information: Table 3). This is further supported by the observation that the purified PINO, one of the main lignans in “callus” wood rosins, enhanced keratinocyte proliferation, similar to the RE substance.

Resin acids are the main ingredients in natural coniferous wood rosins in general, in both “callus” origin and industrial extracts of the coniferous tall oil.^1^ In aqueous RE or RO dispersions, resin acids occur at concentrations of 150 ppm at maximum and appear as oxidized forms of dehydroabietic acid (see Table 1). According to the present observations, these resin acids do not stimulate keratinocytes to proliferate. This is the case with PCA as well. On the other hand, even though not being keratinocyte stimulators, neither resin acids nor PCA caused observable cell death. Even in experiments lasting up to 6 days, the keratinocyte growth in culture with RO and PCA was equal to those with control cultures.

### 4.3 Expression of proinflammatory cytokines in PBMCs

Wound healing can be divided into four phases: hemostasis phase, inflammatory phase, proliferative phase, and remodeling phase. Re‐epithelization takes place during the proliferative phase and the scar starts to mature at the remodeling phase. Each phase is necessary for the next phase to begin. Chronic inflammation in diabetic wounds results in a reduction in the proliferation and further reduced migration of keratinocytes, endothelial cells, and fibroblasts needed to repair the wound.

TNF‐α, IL‐6, IL‐8, and IL‐1β are proinflammatory cytokines that are upregulated during the inflammatory phase of wound healing. They are predominantly produced by monocytes and macrophages in the wound site and are known to play a role in the proliferation of fibroblasts and keratinocytes.^14^, ^15^ These cytokines modulate the inflammatory and reparative process and are involved in the differentiation, activation, and proliferation of many cells such as leukocytes, endothelial cells, keratinocytes, and fibroblasts.^16^, ^17^ In recent studies, triterpene extracts have been shown to elevate the IL‐6 and IL‐8 levels in human primary keratinocyte cultures.^18^

TNF‐α is a multifunctional cytokine that plays a very diverse role in human wound healing, participating in immune system homeostasis, antimicrobial defense, regulation of apoptosis, cell proliferation, and differentiation. The effects of exogenous TNF‐α are dependent on concentration and duration of exposure emphasizing the importance of balancing the proinflammatory signals controlling wound healing. TNF‐α, at low levels, at the early‐stage inflammation, can promote wound healing by indirectly stimulating inflammation, whereas at higher levels, and at a prolonged inflammation, TNF‐α may contribute to the slow healing of the wound.^19^ In high glucose conditions, TNF‐α has been shown to upregulate TIMP1 expression in keratinocytes resulting in impaired keratinocyte migration, and the targeting of TNF‐α has been suggested as a potential therapy to improve diabetic wound healing.^20^

Elevated levels of IL‐1β have a similar effect to that of TNF‐α. They have been shown to maintain each other's expression and therefore amplify this signal.^21^ TNF‐α, as well as IL‐1β, induced keratinocyte activation plays a pivotal role in the pathogenesis of many inflammatory skin diseases, such as psoriasis, and different inhibitors of these cytokines have been proven to be a promising treatment in such diseases.^22^ Both TNF‐α and IL‐1β have been shown to be key mediators of inflammation maintaining a positive feedback loop that sustains inflammation in the diabetic wound.^23^

The in vitro tests with PBMCs were set up to investigate whether the present test articles have any influence on the expression of common proinflammatory cytokines in human blood mononuclear cells in validated tests. As compared to controls and blanks, all present test articles showed observable changes in the expression of cytokines in a way that cannot be explained by simple cytotoxic effects. The observed changes differed markedly from controls in a consistent manner. Expression of TNF‐α decreased significantly and consistently with RE and PINO, while with other test articles the effect was less pronounced. A similar phenomenon was also observed with IL‐1β. On the other hand, the expression of IL‐6 was constantly high with RE and PINO, but to a lesser extent with PCA and low with RO. PINO was the only test article that also promoted the expression of IL‐8, although the increase remained moderate.

The expression of IL‐6 after exposure to PINO or RE was markedly high and remained elevated in similar test conditions in which a significant decrease occurred in the expression of TNF‐α with PINO or RE. These observations suggest that PINO is the component among the present test articles that causes the differences in the observed cytokine expressions, particularly in effects caused by RE.

Resolain™ is a commercially available scalp tonic that has passed safety tests according to the EU Medical Device regulation. Although Resolain™ appeared to strongly inhibit the expression of all cytokines tested, some degree of cytotoxic effect cannot be completely excluded in this particular test setup due to its significantly higher extract concentration.

## 5 CONCLUSIONS

The novel aqueous dispersions of natural “callus” wood rosin from injured trunks of Norway spruce trunks enhance the proliferation of human keratinocytes in vitro. In these dispersions, PINO and presumably lignans, in general, are the main components producing keratinocyte proliferation and uphold. In addition, lignans, as exemplified by PINO in the present in vitro test, seem to influence the expression of cytokines present in human inflammatory cells that are likely relevant to the biology of human skin growth and renewal.

Chronic skin ulcers, including diabetic ulcers, venous ulcers, and pressure ulcers are major clinical challenges. They can result in complete loss of epidermal cells in the ulcerated area, which makes them extremely difficult to treat. Resins and resin‐based salves and ointments have been used in the treatment of acute and chronic wounds for centuries. However, resin‐induced contact dermatitis, which is observed in 2–4 of the human population, limits the clinical application of these products. By downregulating the expression of TNF‐α, while promoting the proliferation of keratinocytes, PINO may provide a potent new therapy in the treatment of chronic wounds.

## AUTHOR CONTRIBUTIONS

**Elias Haapakorva**: Conceptualization; data curation; formal analysis; investigation; methodology; visualization; writing – original draft; writing – review and editing. **Hannu Raunio**: Data curation; supervision; validation; writing – review and editing. **Atte von Wright**: Data curation; supervision; validation; writing – review and editing. **Ilkka Harvima**: Methodology; project administration; resources; supervision; validation; writing – review and editing.

## CONFLICT OF INTEREST

The cytokine release assays were sponsored by, and the test articles were provided by, Repolar Pharmaceuticals Ltd. The research may lead to the development of new products, in which the authors have no business and/or financial interest. The stated financial relationships had no involvement in the study design or in the decision to submit the report for publication.

## ETHICS STATEMENT

This work has been conducted in accordance with Wiley's Best Practice Guidelines on Publishing Ethics and it has been performed in an ethical and responsible way, with no research misconduct, which includes, but is not limited to data fabrication and falsification, plagiarism, image manipulation, unethical research, biased reporting, authorship abuse, redundant or duplicate publication, and undeclared conflicts of interest.

## TRANSPARENCY STATEMENT

The lead author Elias Haapakorva affirms that this manuscript is an honest, accurate, and transparent account of the study being reported; that no important aspects of the study have been omitted; and that any discrepancies from the study as planned (and, if relevant, registered) have been explained.

## Supporting information

Supporting information.

## Data Availability

The data that support the findings of this study are available from the corresponding author upon reasonable request.
