# Supplementary material for: Pinoresinol stimulates keratinocyte proliferation and downregulates TNF‐α secretion in peripheral blood mononuclear cells: An experimental in vitro study
Source: Health Sci Rep. 2022 Dec 16;6(1):e998. doi: 10.1002/hsr2.998 (PMC9758476; doi:10.1002/hsr2.998)
Supplement: Supplementary file 1 — Supporting information. [file HSR2-6-e998-s001.docx]

**14. APPENDICES**

**Figure 4 A-D.** Effect of the test articles on TNFα (A), IL-1β (B), IL-6 (C) and IL-8 (D) release from human PBMCs from 42 years old female donor stimulated with LPS (100 ng/ml). Cytokine levels were measured at 18 hours after the LPS induction. Data is expressed as percentage of the vehicle control groups (cells receiving vehicle and LPS) and shown as mean +/- SD from triplicate wells. The “no-LPS” wells represent the basal release of cytokine from unstimulated cells. Statistical analysis between data sets was carried out by one-way ANOVA followed by Dunnett’s post-test. *** P<0.001 denotes significant cytokine release reduction compared to control. ^###^ P<0.001, ^#^ P<0.05 denotes significant elevation of cytokine release compared to control.

**Table 3.** The effects of RE, RO, PCA and PINO on the proliferation of human primary keratinocytes in 2 or 6 days in high-calcium (DMEM) or low-calcium (KSFM) conditions with (+) or without (-) added growth stimulators.

| **Medium** |  | |  | **KSFM** | | | **KSFM** | | | | **DMEM** | | | **DMEM** | | |
| --- | --- | --- | --- | --- | --- | --- | --- | --- | --- | --- | --- | --- | --- | --- | --- | --- |
| **Gr. Stim.** |  | |  | **+** | | | **-** | | | | **+** | | | **-** | | |
| **Time (d)** | **TA** | **Dil. (%)** | | **Mean** | **SD** | **Sig.** | **Mean** | **SD** | **Sig.** | **Mean** | | **SD** | **Sig.** | **Mean** | **SD** | **Sig.** |
| 2 | RE | 4.0 | | 132.2 | 9.840 | *** | 160.9 | 4.741 | *** | 127.08 | | 7.19 | *** | 123.3 | 5.230 | ** |
| 2 | RE | 2.0 | | 128.3 | 13.042 | *** | 141.1 | 12.486 | *** | 133.01 | | 10.18 | *** | 155.2 | 17.689 | *** |
| 2 | RE | 1.0 | | 99.7 | 1.226 |  | 116.0 | 7.667 |  | 108.16 | | 5.95 |  | 101.0 | 5.532 |  |
| 2 | RE | 0.5 | | 109.2 | 4.065 | * | 105.3 | 7.375 |  | 109.80 | | 6.58 | * | 106.5 | 10.269 |  |
| 2 | RO | 4.0 | | 92.0 | 2.465 |  | 103.6 | 3.334 |  | 98.33 | | 6.94 |  | 92.9 | 5.236 |  |
| 2 | RO | 2.0 | | 94.0 | 11.967 |  | 105.7 | 4.732 |  | 98.08 | | 8.02 |  | 97.1 | 8.019 |  |
| 2 | RO | 1.0 | | 86.5 | 1.812 |  | 95.3 | 2.324 |  | 96.41 | | 3.91 |  | 104.3 | 5.411 |  |
| 2 | RO | 0.5 | | 95.2 | 2.578 |  | 112.8 | 8.629 |  | 104.21 | | 5.48 |  | 117.2 | 9.913 | * |
| 2 | PCA | 4.0 | | 91.6 | 1.124 |  | 97.8 | 2.979 |  | 94.50 | | 2.08 |  | 93.3 | 3.858 |  |
| 2 | PCA | 2.0 | | 88.9 | 3.055 |  | 97.4 | 6.048 |  | 97.68 | | 7.61 |  | 98.1 | 4.191 |  |
| 2 | PCA | 1.0 | | 90.7 | 2.297 |  | 95.2 | 4.139 |  | 100.99 | | 10.31 |  | 109.8 | 4.625 |  |
| 2 | PCA | 0.5 | | 94.5 | 5.453 |  | 97.2 | 4.122 |  | 104.49 | | 3.43 |  | 119.9 | 6.333 | * |
| 2 | PINO | 4.0 | | 110.3 | 2.866 |  | 142.5 | 5.094 | *** | 132.23 | | 10.10 | *** | 139.5 | 6.893 | *** |
| 2 | PINO | 2.0 | | 110.2 | 2.906 |  | 128.9 | 5.940 | *** | 130.95 | | 11.81 | *** | 182.4 | 14.802 | *** |
| 2 | PINO | 1.0 | | 97.1 | 0.917 |  | 127.2 | 3.747 | *** | 121.30 | | 9.58 | *** | 108.0 | 4.386 |  |
| 2 | PINO | 0.5 | | 101.3 | 2.886 |  | 114.5 | 7.693 | * | 114.15 | | 11.63 | ** | 106.0 | 4.690 |  |
| 2 | SECO | 4.0 | | 89.0 | 1.279 |  | 112.9 | 7.569 |  | 102.9 | | 4.336 |  | 101.4 | 2.338 |  |
| 2 | SECO | 2.0 | | 97.7 | 2.310 |  | 107.5 | 9.089 |  | 99.3 | | 6.810 |  | 100.3 | 4.882 |  |
| 2 | SECO | 1.0 | | 91.6 | 2.843 |  | 103.1 | 5.787 |  | 100.8 | | 6.428 |  | 102.5 | 3.148 |  |
| 2 | SECO | 0.5 | | 98.0 | 2.706 |  | 103.2 | 6.082 |  | 100.1 | | 9.494 |  | 102.9 | 6.137 |  |
| 2 | NTG | 4.0 | | 88.1 | 1.265 |  | 100.2 | 5.336 |  | 98.7 | | 3.252 |  | 104.2 | 2.031 |  |
| 2 | NTG | 2.0 | | 98.5 | 2.916 |  | 100.6 | 7.784 |  | 94.5 | | 4.876 |  | 101.3 | 5.113 |  |
| 2 | NTG | 1.0 | | 92.0 | 2.202 |  | 92.5 | 2.864 |  | 101.7 | | 5.445 |  | 105.4 | 3.420 |  |
| 2 | NTG | 0.5 | | 97.5 | 2.180 |  | 94.9 | 4.297 |  | 99.3 | | 6.213 |  | 106.6 | 4.922 |  |
| 6 | RE | 4.0 | | 137.1 | 7.304 | *** | 167.6 | 11.931 | *** | 188.0 | | 6.536 | *** | 116.1 | 4.885 | *** |
| 6 | RE | 2.0 | | 132.8 | 10.836 | *** | 120.2 | 12.710 |  | 165.8 | | 13.082 | *** | 124.9 | 4.687 | *** |
| 6 | RE | 1.0 | | 99.1 | 4.491 |  | 102.8 | 1.831 |  | 120.2 | | 6.652 | ** | 99.0 | 4.610 |  |
| 6 | RE | 0.5 | | 103.5 | 5.108 |  | 88.1 | 9.545 |  | 107.8 | | 5.548 |  | 101.6 | 6.436 |  |
| 6 | RO | 4.0 | | 62.8 | 3.135 | ^###^ | 72.9 | 4.969 | ^#^ | 91.8 | | 4.841 |  | 92.5 | 4.887 |  |
| 6 | RO | 2.0 | | 91.4 | 6.508 |  | 102.2 | 6.689 |  | 89.7 | | 5.495 |  | 95.4 | 3.498 |  |
| 6 | RO | 1.0 | | 79.7 | 5.294 | ^#^ | 83.9 | 1.638 |  | 88.7 | | 6.257 |  | 92.7 | 5.350 |  |
| 6 | RO | 0.5 | | 113.8 | 7.251 |  | 123.6 | 12.807 |  | 93.2 | | 5.639 |  | 93.1 | 3.759 |  |
| 6 | PCA | 4.0 | | 76.9 | 3.205 | ^#^ | 74.5 | 1.868 |  | 84.5 | | 7.323 | ^#^ | 93.0 | 2.739 |  |
| 6 | PCA | 2.0 | | 94.7 | 8.793 |  | 99.4 | 8.660 |  | 86.9 | | 7.687 |  | 91.6 | 5.190 |  |
| 6 | PCA | 1.0 | | 88.9 | 2.799 |  | 114.6 | 12.430 |  | 94.5 | | 3.689 |  | 97.2 | 3.936 |  |
| 6 | PCA | 0.5 | | 111.1 | 10.740 |  | 131.9 | 8.219 | ** | 97.6 | | 2.829 |  | 98.3 | 5.362 |  |
| 6 | PINO | 4.0 | | 108.0 | 7.360 |  | 181.8 | 4.881 | *** | 206.7 | | 3.040 | *** | 156.7 | 7.151 | *** |
| 6 | PINO | 2.0 | | 112.6 | 8.203 |  | 195.7 | 10.487 | *** | 200.0 | | 12.318 | *** | 154.7 | 8.397 | *** |
| 6 | PINO | 1.0 | | 100.0 | 7.537 |  | 171.2 | 7.999 | *** | 144.2 | | 8.283 | *** | 122.6 | 4.972 | ** |
| 6 | PINO | 0.5 | | 99.8 | 5.443 |  | 161.9 | 5.937 | *** | 128.6 | | 9.241 | *** | 127.0 | 8.307 | *** |
| 6 | SECO | 4.0 | | 74.7 | 4.778 | ^###^ | 136.4 | 6.935 | *** | 99.7 | | 7.412 |  | 95.1 | 2.671 |  |
| 6 | SECO | 2.0 | | 92.4 | 2.614 |  | 134.4 | 11.616 | ** | 99.2 | | 6.672 |  | 100.4 | 10.699 |  |
| 6 | SECO | 1.0 | | 99.0 | 5.251 |  | 146.1 | 8.780 | *** | 97.4 | | 7.801 |  | 95.8 | 4.445 |  |
| 6 | SECO | 0.5 | | 106.0 | 5.538 |  | 158.4 | 8.924 | *** | 103.2 | | 5.813 |  | 94.6 | 17.403 |  |
| 6 | NTG | 4.0 | | 80.7 | 4.348 | ^#^ | 97.0 | 4.137 |  | 93.4 | | 6.307 |  | 90.3 | 5.468 |  |
| 6 | NTG | 2.0 | | 105.6 | 4.339 |  | 119.7 | 6.112 |  | 93.2 | | 2.402 |  | 97.2 | 7.261 |  |
| 6 | NTG | 1.0 | | 87.4 | 3.655 |  | 142.9 | 7.781 | *** | 101.9 | | 6.038 |  | 98.2 | 4.973 |  |
| 6 | NTG | 0.5 | | 102.0 | 2.618 |  | 139.3 | 6.200 | *** | 96.6 | | 8.053 |  | 94.9 | 7.417 |  |

**Note:** Values represent mean absorbances of five replicates +/- SD, normalized to vehicle control (100). *** P<0.001, ** P<0.01, and * P<0.05 denotes significant increase in measured absorbance compared to control. ^###^ P<0.001, ^##^ P<0.01, and ^#^ P<0.05 denotes significant decrease in measured absorbance compared to control. TA = test article, RE = resin, RO = rosin, PINO = pinoresinol, SECO = secoisolariciresinol, NTG = nortrachelogenin.
